# Low internal pressure in femtoliter water capillary bridges reduces evaporation rates

**DOI:** 10.1038/srep22232

**Published:** 2016-03-01

**Authors:** Kun Cho, In Gyu Hwang, Yeseul Kim, Su Jin Lim, Jun Lim, Joon Heon Kim, Bopil Gim, Byung Mook Weon

**Affiliations:** 1 Soft Matter Physics Laboratory, School of Advanced Materials Science and Engineering, SKKU Advanced Institute of Nanotechnology (SAINT), Sungkyunkwan University, Suwon 440-746, Korea; 2 Beamline Division, Pohang Light Source, Hyoja, Pohang, Kyung-buk, 790-784, Korea; 3 Advanced Photonics Research Institute, Gwangju Institute of Science and Technology (GIST), Gwangju, 500-712, Korea; 4 Department of Bio and Brain Engineering, Korea Advanced Institute of Science and Technology (KAIST), Daejeon, 305-701, Korea

## Abstract

Capillary bridges are usually formed by a small liquid volume in a confined space between two solid surfaces. They can have a lower internal pressure than the surrounding pressure for volumes of the order of femtoliters. Femtoliter capillary bridges with relatively rapid evaporation rates are difficult to explore experimentally. To understand in detail the evaporation of femtoliter capillary bridges, we present a feasible experimental method to directly visualize how water bridges evaporate between a microsphere and a flat substrate in still air using transmission X-ray microscopy. Precise measurements of evaporation rates for water bridges show that lower water pressure than surrounding pressure can significantly decrease evaporation through the suppression of vapor diffusion. This finding provides insight into the evaporation of ultrasmall capillary bridges.

Liquid capillary bridges are ubiquitous in microscopic or nanoscopic confinement. In general, capillary bridges are thermodynamically formed through capillary condensation, which is quantified by the Kelvin equation. They are also hydrodynamically formed during the evaporation process in a confined location between two surfaces. In this work, we visualized evaporating water capillary bridges between a microsphere and a flat solid surface with X-ray microscopy. Microscopic capillary bridges have attracted much attention because they exhibit interesting kinetics^1^, showing either capillarity-induced condensation or evaporation, which impacts many processes such as ink deposition, spray drying, evaporative lithography, and fluid dynamics in powders^2^. Capillary forces exerted by Laplace pressure in capillary bridges play essential roles in the bonding of microscale or nanoscale objects^3^. Particularly, in several natural and industrial situations, water bridges frequently appear and mediate physical phenomena: for instance, adhesion between grains^4^,^5^, suspension rheology^6^,^7^, sand friction^8^, capillary interactions^3^,^9^, adhesion between soft substrates^10^, and particle indentation into tissues^11^. The formation and interpretation of capillary bridges are significant operations in atomic force microscopy^12^,^13^. Furthermore, liquid bridges at nanoscale contacts^14^ usually cause capillarity-induced negative pressure inside the bridges^15^,^16^,^17^. For instance, negative pressure in liquid capillary bridges can reach −160 MPa for a very small volume around the AFM tip radius (e.g., ~10 nm)^17^. Negative pressure inside nanoscale liquid bridges can modify classical phase transitions according to the scale^18^, including the evaporation kinetics^19^. Evaporation of femtoliter (1 fL = 1 μm^3^) sessile droplets that have positive internal pressure larger than 1 atm has been studied experimentally^20^,^21^,^22^ and numerically^23^. (See a recent review^24^.) Recent studies^20^,^24^ suggest that the evaporation kinetics of ultrasmall volumes with internal pressure larger than 1 atm can deviate from macroscopic laws for droplet evaporation^25^,^26^,^27^. Detailed understanding of the rapid evaporation process in femtoliter water drops is important for controlling evaporation kinetics in femtoliter chemistry, where stable femtoliter containers are under development for chemical and biochemical analysis^28^,^29^. The influence of internal pressure lower than 1 atm on the evaporation kinetics of femtoliter capillary bridges remains unclear. There is a lack of experiment-based understanding on the detailed evaporation kinetics of femtoliter capillary bridges with negative pressure, with the exception of a few numerical studies^19^,^30^. High-resolution visualization of liquid surface profiles is necessary to figure out the unique dynamic features of capillary bridges^31^,^32^. A technique applicable to microscale or nanoscale objects that can provide direct, real space, and real-time visualization is still required to precisely quantify the evaporation kinetics of capillary bridges for ultrasmall liquids.

In this paper, we present a feasible experimental method to directly quantify the evaporation kinetics of water capillary bridges surrounding a microsphere on a flat solid surface by precisely tracking the water surface profiles with transmission X-ray microscopy, as demonstrated and illustrated in Figs 1 and 2. This imaging technique enables us to evaluate the evaporation rate of a femtoliter water bridge. Particularly, we find that internal pressure lower than 1 atm inside the bridge can significantly reduce the evaporation rates.

## Results

Precise quantitative analyses for changes in meniscus profiles and volumes of water capillary bridges were performed. We observed the two evaporation stages and measured the evaporation rates at the final stages.

### Pinning-Depinning Transition

Thanks to the accurate tracking of water surface profiles, we are able to observe two distinct stages in the evaporation dynamics of the water film surrounding a single microsphere. As demonstrated in Fig. 3(a), the water film height *h*, which is normalized by the particle diameter *d*, changes with time as the water film initially gets pinned and then quickly depinned during evaporation. The sudden-death-like event from pinning to depinning occurs at the late stages close to the final evaporation time for each event. To analyze this behavior, we utilize a standard method that involves rescaling time by the characteristic time *α*, taken at *h*/*d* = exp (−1) for each curve. The characteristic time is usually adopted to measure the lifetime of a decay curve, enabling us to reconcile the different final evaporation times for each event. Such rescaling in Fig. 3(b) reveals that the first pinning stage appears up to *t*/*α* ~ 0.96, corresponding to *θ* ~ 60^o^, which is interestingly equivalent to *a* ≈ ½ *r*_*p*_ in Fig. 2(a). In addition, the second depinning stage emerges for a very short time: the main sudden change occurs around *t* ~ *α*. On the other hand, the water contact angle *ϕ* defined from the three-phase contact line for water-air-particle interfaces increases at the pinning stage, *t* < *α*, as the height of the water surface is gradually decreased by pinning at the three-phase contact line and quickly decreases to zero at the depinning stage, around *t* ~ *α*, as demonstrated in Fig. 3(c). The abrupt decrease of the contact angle at the final stage is due to the depinning process and the hydrophilicity of the functionalized microspheres. The origin of pinning might be related to the heterogeneity of the particle surface or the non-equilibrium state of the water bridge due to evaporation. The microspheres used here are almost monodispersed, *r*_*p*_ = 3.0 ± 0.03 μm, as demonstrated by the SEM image in Fig. 3(d), hence, our experiments were robustly repeatable. At the final stage, the water contact angle is almost zero and the small volume liquid meniscus is catenoidal. This meniscus belongs to a capillary bridge between a sphere and a flat surface, which is a three-dimensional object, and its cross-sectional image looks circular. As a result, the water bridge approximately becomes a catenoidal capillary bridge that has a circular radius *R*.

### Evaporation Rates

Precise measurements of time-dependent small volumes for water catenoidal capillary bridges at the depinning stage provide quantitative information about the evaporation kinetics for femtoliter capillary bridges. As shown in Fig. 4, during the final evaporation stage for water capillary bridges, the water volumes gradually decrease with time from the characteristic volumes *v*_0_ ~ 240 fL that approximate the volume at *t* ~ *α*. Here, we modify a general evaporation equation for a spherical cap model^20^,^24^ to describe the evaporation equation for a catenoidal capillary bridge. The water volume changes at a given moment for capillary bridges may be expressed as


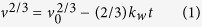


where *k*_*w*_ is an experimental constant that does not depend on time and is evaluated as ~248 fL^2/3^/s for water. The measured *k*_*w*_ value can be used to evaluate how much a calibrated capillary bridge evaporation deviates from a typical sessile drop evaporation. This *v*^2/3^ versus *t* relation is natural in sessile drop evaporation^20^,^24^ and *v* → 0 as *t* → *t*_*f*_ (the complete evaporation time). The evaporation rate is then determined by differentiating the above Eq. (1) as


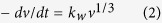


The evaporation rate would be proportional to the circular radius *R* of the water bridge at the final stage of evaporation (Fig. 4). (R can be measured during ~0.12s prior to *t*_*f*_).

## Discussion

We identified the existence of two evaporation stages for a water film surrounding a microsphere, as demonstrated in Fig. 3(b). This behavior is similar to the wetting-drying transition^36^. The pinning of a water film on the surface of a microsphere would be attributed to the surface heterogeneities of the microsphere, which would affect the dynamic wetting behavior^37^,^44^,^45^ and therefore, the consequential evaporation kinetics. The two-stage evaporation dynamics is known^2^,^42^ but direct visualization of the two stages and the effect of low internal pressure on the evaporation rate are studied in this work.

Figure 5 illustrates the pinning-depinning critical condition at the TCL: the vertical force components would be balanced as (*γ*_*PA*_ − *γ*_*PW*_) sin*θ* = *γ*_*WA*_ sin(*θ − ϕ*) for the interfacial tensions (*γ*) among the particle (_*P*_), air (_*A*_), and water (_W_). This situation can be valid only if the upper part of the TCL is dry, which would be possible during the first pinning stage. Taking *γ*_*PA*_ − *γ*_*PW*_ ≈ 46 mN/m and *γ*_*WA*_ = 72 mN/m for polystyrene, air, and water^46^, the vertical force balance leads to the pinning condition for *ϕ* ≈ 26.4^o^ at *θ* = 60^o^ (equivalently *a* ≈ ½ *r*_*p*_), which is consistent with our observation *ϕ* ≈ 30^o^ in Fig. 3(c). A recent simulation work reported the same condition *a* ≈ ½ *r*_*p*_ for pinning at similar conditions^47^.

At the depinning stage around *t* ~ *α*, the water bridges are catenoidal, as demonstrated in Fig. 4 (the inset) and supported by the almost zero water contact angle *ϕ* in Fig. 3(c). The *R* dependence of the evaporation rate for a water capillary bridge is similar to the empirical evaporation law for microscopic sessile drops 24,25,26,27. This scaling suggests that the evaporation rate is proportional to the perimeter of the droplet. The –*dv*/*dt* ∝  *R* scaling would be related to the vapor diffusion mechanism, which can be described as *dv*/*dt* ∝ *R*^2^*D*(*dc*/*dR*), where *D* is the diffusivity of the vapor, *c* is the concentration of the vapor^24^,^26^, and *R*^2^ originates from the surface area of the bridge.

Despite the different geometry, consideration of the evaporation rate between a bridge and a hemisphere sessile drop with an identical surface area is useful. The surface area of the water bridge is estimated to be ~100 μm^2^ for *v*_0_ ~ 240 fL and is equivalent to the surface area of a hemisphere sessile drop with a radius ~4.0 μm. The measured evaporation rate of 1.5 pL/s at *v*_0_ ~ 240 fL in Fig. 4 is much smaller than the estimate of 8.9 pL/s for a 4.0-μm-radius hemisphere sessile drop, based on the Hu-Larson evaporation equation^27^ -*dm*/*dt* = *πRD* (1 - *H*)*c*_*v*_ (0.27*θ*^2^ + 1.30) (where *H* is the relative humidity, *c*_*v*_ the saturated water vapor concentration, and *θ* the contact angle) calculated by adopting normal conditions for water at room temperature and 33.5% relative humidity. The Kelvin equation enables us to estimate the relative vapor pressure, which is given as ~0.9998 and is thus negligible for our case (by using material constants for water^14^,^15^,^16^,^17^,^18^). This consideration suggests that femtoliter catenoidal capillary bridges can have lower internal pressure than 1 atm and thus exhibit slower evaporation rates than sessile drops with identical surface areas.

The slow evaporation rate for the catenoidal water bridge is attributed to the lower internal pressure of the bridge. The pressure inside the bridge *p*_*w*_ is estimated to be 0.6 atm for *v*_0_ ~ 240 fL, and 1.2 atm for the equivalent-surface-area hemisphere drop. (The estimation model of *p*_*w*_ was adapted from refs. 17,18). Here slow evaporation from the water bridge is plausible through suppression of the vapor diffusion from 0.6 to 1.0 atm (to the surrounding pressure), compared with the evaporation from 1.2 to 1.0 atm for the hemisphere sessile drop. This estimation implies that lower liquid pressure than the surrounding atmospheric pressure would cause a significant reduction in the evaporation rate at femtoliter scales, as qualitatively suggested^1^,^31^.

Our experiment shows that lower or negative internal pressure may cause a decrease in the evaporation rate. Other factors could also play a role in the reduction of the capillary evaporation rate. For example, the geometry of a sessile droplet can increase the evaporation rate at the edge^48^. There may be the possibility of a geometric effect in which the vapor diffusion is hindered by the microsphere and the flat substrate. The catenoidal capillary bridge shape would be important for reducing the evaporation rate. After the liquid molecule enters the area just above the liquid-vapor interface and moves around, if it moves in the direction normal to the interface, it may have a greater likelihood of diffusing into vapor. However, when it moves in other directions, it would be more likely to be captured by the liquid since the liquid surface is curved instead of flat. On the other hand, at very late stages (*t* − *t*_*f*_ ≈ 0), the competition between capillary pressure and disjoining pressure might have a significant effect on the evaporation kinetics: disjoining forces significantly dominate capillary forces in ultra-thin films^19^,^49^,^50^. Consequently, the quantitative evaporation kinetics for capillary bridge evaporation is quite predictable to engineers. Additionally, the presence of low or negative liquid pressure, *p*_*w*_ < 1.0 atm, at femtoliter scales can suppress vapor diffusion from the bridge surface to the atmosphere.

Finally, we emphasize the feasibility of transmission X-ray microscopy for exploring in detail the evaporation kinetics of femtoliter water capillary bridges. The meniscus could be extracted from X-ray microscopy and Image Pro-Plus software. This technique could provide a high spatial resolution of 46 nm, which is enough for direct visualization of capillary bridge evaporation in still air between a microsphere and a flat solid surface. Better resolution from X-ray microscopy is expected and thus further useful study is promising for hydrodynamics study on capillary bridges. Detailed information on the evaporation kinetics of capillary bridges in microscopic or nanoscopic confinement would be useful in many natural and industrial situations. At femtoliter scales, capillary bridges are well known to have lower internal pressure than the ambient surrounding pressure. Our finding shows that lower internal pressure than the surrounding pressure can significantly decrease evaporation through the suppression of vapor diffusion. This finding provides a clue for better understanding of the evaporation of ultrasmall capillary bridges.

## Methods

X-ray microscopy is a promising *in-situ* technique in soft matter research^33^. We utilize the full-field transmission X-ray microscopy at the 7C X-ray Nano Imaging (XNI) beamline established at the Pohang Light Source II^34^. This beamline is dedicated primarily to full-field imaging of the internal structures of biological and material samples. The beamline provides 46 nm resolution with a 32.7 × 15.3 μm^2^ field of view. A focused hard X-ray beam with 6.717 keV photon energy from a synchrotron source and a high speed camera (pco. dimax S4; 25 fps, 2016 × 944 pixels) were used to acquire real-time evaporation dynamics. Then 6.0-μm-diameter polystyrene (Catalog No. 07312-5, Polysciences, Inc.) microspheres suspended in pure water were used for evaporation experiments. The polystyrene in the core of the microspheres was intrinsically hydrophobic, and we used anionic sulfate functionalized polystyrene microspheres that are hydrophilic and stably suspended in water. A droplet of the microsphere suspension was gently placed on the top of a flat solid surface made of borosilicate glass tubes (GC-1, Narishige Co., Japan) with outer diameter and thickness of 1.0 and 0.2 mm, respectively. X-ray microscopy was used to obtain sequential side-view images for the evaporation process of the microsphere suspension on the flat solid surface. The detailed principle and the setup of X-ray microscopy can be seen in refs. 33,34. X-ray imaging technique is an elegant *in-situ* visualization method for clear differentiation of water, air, and particle interfaces.

**Figure 1:**
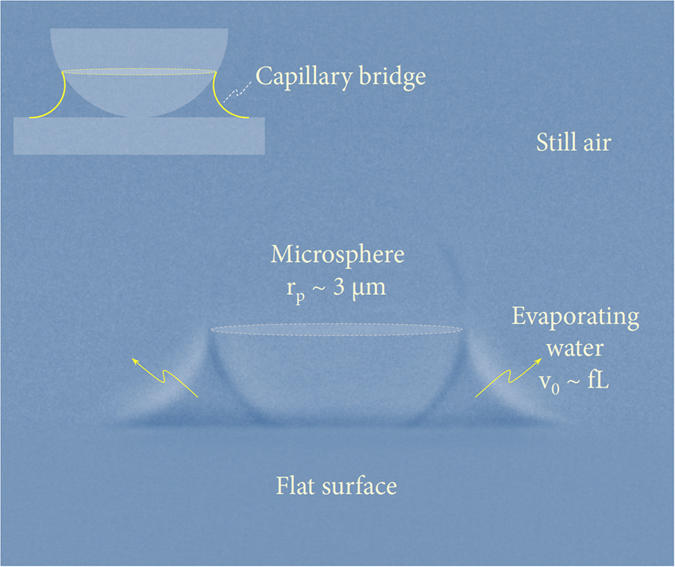
Visualizing capillary bridges. Clear visualization of a water capillary bridge between a microsphere on a flat solid surface, taken by high-resolution transmission X-ray microscopy. A water film that is thicker than the microsphere diameter gradually evaporates in still air in the early stages and eventually evolves into a circular capillary bridge surrounding a microsphere with a radius *r*_*p*_ ~ 3 μm on a flat substrate at late stages. The water bridge has a small volume of ~100 fL taken from an image at 0.12 seconds prior to complete evaporation in the final image (see Supplementary Movies 1 and 2).

Representative evidence is provided in Fig. 1 to demonstrate clear visualization of the water capillary bridge between a microsphere on a flat solid surface, obtained with high-resolution transmission X-ray microscopy. A water film that is thicker than the microsphere diameter gradually evaporates in still air at the early stages and eventually evolves into a catenoidal capillary bridge surrounding a polystyrene microsphere with a radius *r*_*p*_ ~ 3 μm on a flat glass substrate at late stages (Fig. 1). The water bridge has a small volume of ~100 fL taken from an image at 0.12 seconds prior to complete evaporation in the final image.

**Figure 2:**
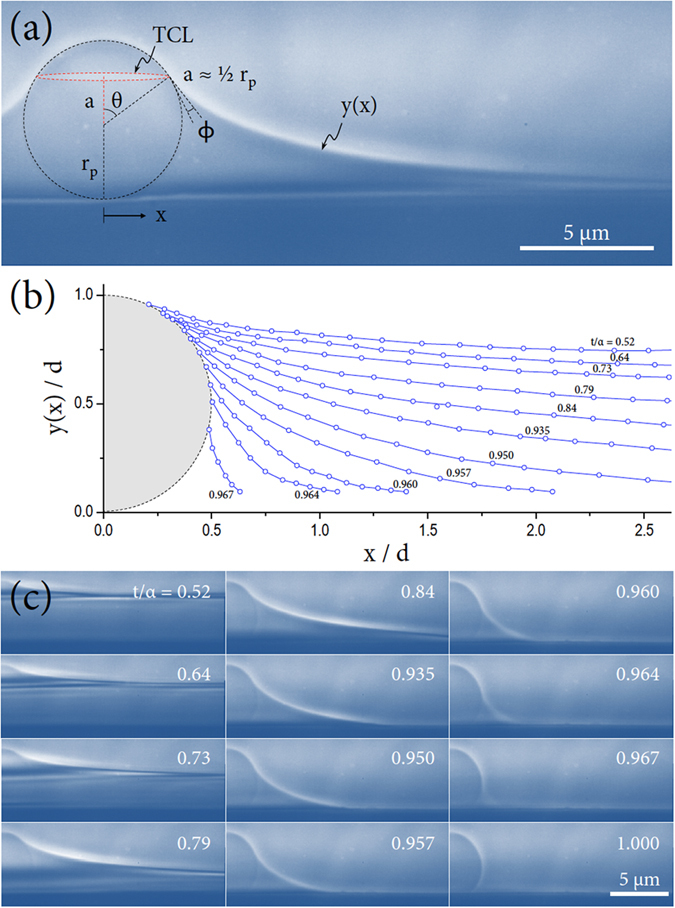
X-ray microscopic observations. (**a**) A representative image for the surface profiles of an evaporating water film surrounding a single microsphere on a flat surface, taken by high-resolution transmission X-ray microscopy, shows clear boundaries among air, water, particle, and substrate. Particularly, this image shows the moment when the water surface was pinned on the particle surface at a condition, *a* ≈ ½ *r*_*p*_, where the length *a*, given by *a* = *r*_*p*_ cos*θ*, is related to the water film height *h* = *r*_*p*_ + *a*. The water contact angle *ϕ* is associated with a dynamic force balance at the three-phase contact line (TCL) for the particle, water, and air^11^. (**b**) The water surface profile *y*(*x*) with position *x*, normalized by the particle diameter *d* = 2*r*_*p*_, were precisely measured by X-ray microscopy. The profiles moved downward during evaporation. Here the profile intervals were arbitrarily chosen to guide the eye. (**c**) The real sequential images for (**b**) (see Supplementary Movie 3).

**Figure 3:**
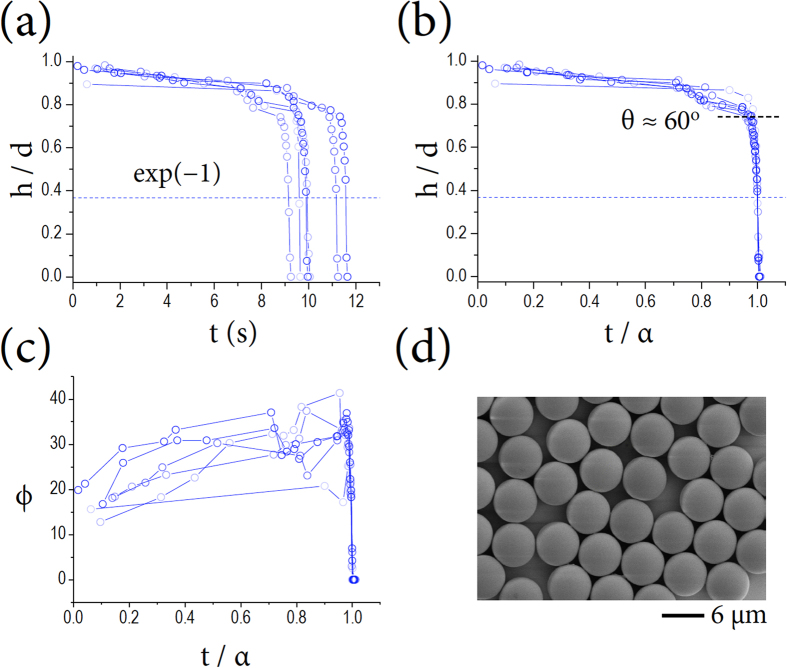
Two evaporation stages. (**a**) The water film height, normalized by each particle diameter, is initially pinned and then quickly depinned by evaporation. This behavior can be analyzed by rescaling time according to the characteristic time *α*, taken at *h*/*d* = exp(−1). (**b**) The first pinning stage appears up to *t*/*α* ~ 0.96, corresponding to *θ* ~ 60^o^, equivalent to *a* ≈ ½ *r*_*p*_. The second depinning stage emerges for a very short time around *t* ~ *α*. (**c**) The water contact angle *ϕ* increases during the pinning stage and quickly decreases up to zero during the depinning stage, indicating that the water bridge becomes catenoidal. (**d**) The microspheres used here are monodispersed, *r*_*p*_ = 3.0 ± 0.03 μm (a standard deviation from 24 datasets), as demonstrated by the SEM image.

**Figure 4:**
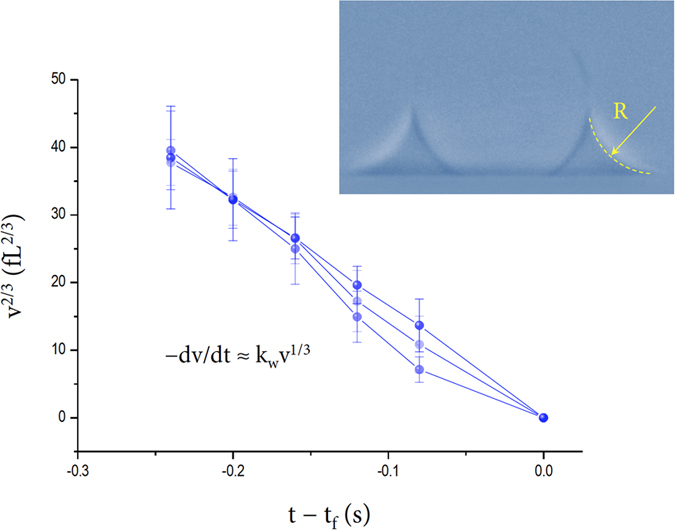
Evaporation rates at the final stages. Evaporation dynamics of water capillary bridges whose volumes gradually decrease with time from characteristic volumes of *v*_0_ ~ 240 fL. The volume changes are described as *v*^2/3^ = *v*_0_^2/3^ – (⅔) *k*_*w*_*t* and *v* → 0 as *t* → *t*_*f*_, where *k*_*w*_ is a constant and is experimentally set as ~ 248 fL^2/3^/s for water. The evaporation rate expression of −*dv/dt* = *k*_*w*_*v*^1/3^ is similar to the sessile drop evaporation law^20^,^24^, but the evaporation rate for a bridge is slower than that for a hemisphere sessile drop with identical surface area. The error bars were determined by the repetition of measurements for each sample.

**Figure 5:**
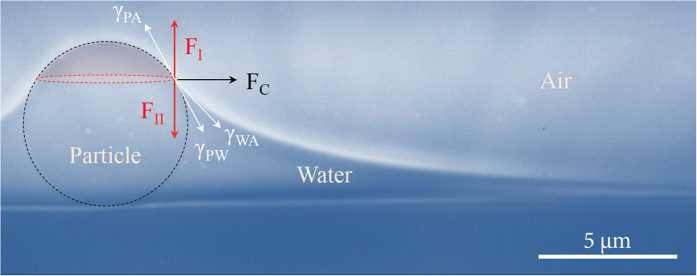
Vertical force balance for contact line pinning. The vertical force components, *F*_*I*_ and *F*_*II*_, is balanced where interfacial tensions (*γ*) exist among the particle (_P_), air (_A_), and water (_W_). The upper part of the TCL is dry during the pinning stage. The horizontal force component *F*_*C*_ contributes to the capillary attraction.

Images were processed with Image Pro-Plus (version 6.0, Media Cybernetics, Inc.). A representative image in Fig. 2(a) shows clear boundaries among air, water, particle, and substrate. Particularly, this image shows the moment when the water surface is pinned on the particle surface at the condition of *a* ≈ ½ *r*_*p*_, where the length *a* is given by *a* = *r*_*p*_ cos *θ* and the water film height can be rewritten as *h* = *r*_*p*_ + *a*. The angle at the interfaces of the particle, water, and air at the contact point is defined as the water contact angle *ϕ*. This angle is associated with a dynamic force balance among the interfacial tensions of the particle, water, and air^11^,^35^,^36^. The water contact angle was actually measured from X-ray sequential images by taking the dynamic contact angle between the tangent lines for particle-water and air-water interfaces at the three-phase contact line (TCL) during the evaporation process, as illustrated in Fig. 2(a). The water surface profile *y*(*x*), which is described with an exponential function and normalized by the particle diameter *d* = 2*r*_*p*_, were precisely measured throughout imaging analysis by X-ray microscopy, as shown in Fig. 2(b). The water surface profiles moved downward during evaporation.

The axisymmetric meniscus profiles were modeled with an exponential function for the middle stages and a catenoidal shape^38^ at the final stages, *t*/*α* > 0.96, instead of the exact complicated expression^39^,^40^. At the final stages of evaporation, the profile was fitted with a circular function for which the coefficient of determination was over 0.99997. The mathematics of the geometry of capillary bridges has been studied in detail analytically^41^−^43^. Precise quantitative analysis for the volume changes of water capillary bridges with time is available.

## Additional Information

**How to cite this article**: Cho, K. *et al.* Low internal pressure in femtoliter water capillary bridges reduces evaporation rates. *Sci. Rep.* **6**, 22232; doi: 10.1038/srep22232 (2016).

## Supplementary Material

Supplementary Information

Supplementary Movie 1

Supplementary Movie 2

Supplementary Movie 3
