# Supplementary material for: Low internal pressure in femtoliter water capillary bridges reduces evaporation rates
Source: Sci Rep. 2016 Mar 1;6:22232. doi: 10.1038/srep22232 (PMC4772007; doi:10.1038/srep22232)
Supplement: Supplementary Information [file srep22232-s1.doc]

**Supplementary Information**

Low internal pressure in femtoliter water capillary bridges reduces evaporation rates

Kun Cho,^1^ In Gyu Hwang,^1^ Yeseul Kim,^1^ Su Jin Lim,^1^ Jun Lim,^2^ Joon Heon Kim,^3^ Bopil Gim,^4^ & Byung Mook Weon^1,*^

**Affiliations:**

^1^ Soft Matter Physics Laboratory, School of Advanced Materials Science and Engineering, SKKU Advanced Institute of Nanotechnology (SAINT), Sungkyunkwan University, Suwon 440-746, Korea

^2^ Beamline Division, Pohang Light Source, Hyoja, Pohang, Kyung-buk, 790-784, Korea

^3^ Gwangju Institute of Science and Technology (GIST), Gwangju, 500-712, Korea

^4^ Department of Bio and Brain Engineering, Korea Advanced Institute of Science and Technology (KAIST), Daejeon, 305-701, Korea

*Corresponding author: bmweon@skku.edu

**Supplementary Video Legends:**

**Supplementary Movie 1:** Clear visualization from event #1 (a sequential movie for **Fig. 1**) of a water capillary bridge between a microsphere on a flat solid surface, taken by high-resolution transmission X-ray microscopy.

**Supplementary Movie 2:** Clear visualization from event #2 (a sequential movie for **Fig. 1**) of a water capillary bridge between a microsphere on a flat solid surface, taken by high-resolution transmission X-ray microscopy.

**Supplementary Movie 3:** Clear visualization from event #3 (a sequential movie for **Fig. 2**) of a water capillary bridge between a microsphere on a flat solid surface, taken by high-resolution transmission X-ray microscopy.
